# An Epidemic of Typhoid, Typhus and Spotted Fever

**Published:** 1865-05

**Authors:** J. T. Pearce

**Affiliations:** Mechanicsburg, O.


### MISCELLANEOUS.

### AN EPIDEMIC OF TYPHOID, TYPHUS AND SPOTTED FEVER.

BY J. T. PEARCE, M. D., MECHANICSBURG, O.

During the past summer and fall, our community enjoyed a remarkable immunity from all varieties of fevers, scarcely a case of well-marked typhoid fever occurring in the entire neighborhood. In the month of October, however, a soldier returned to his home in our town sick of typhoid fever. His case was well defined, of the enteric form, and confined him to his bed and room some six weeks.

Before he had fairly recovered, his mother, a healthy woman, about forty years of age, was taken down with the same variety of disease, next a younger brother, and in quick succession other members of the family were attacked, and, finally, five of the same family were sick of typhoid fever within a few weeks from the recovery of the first case—none escaping but the father, a remarkably stout, healthy man.

From this point of beginning, the disease spread to other families until the majority of the dwellings in that part of the village contained one or more cases of typhoid fever. Gradually it extended to other more distant neighborhoods in town and country, and finally, this was our prevailing form of disease until in the following February, when it merged into, or gave place to several cases of the alarming disease known as Spotted Fever, which at the present writing, March 23d, has I believe subsided, leaving our community again reasonably free from disease.

The disease, from its commencement to its close, selected its subjects alike from all classes of society. Ease and opulence gave no more immunity than squalor and poverty. The only preference it seemed to manifest was for women and children, there being very few adult males affected at any part of the season.

The cases arranged themselves into three varieties, appearing to my mind at least, but separate links of the same chain of disease. First we had the well defined typhoid fever of the enteric variety, next the well marked typhus, and third, the spotted fever, which last form seemed but a rapid evolution of typhoid or typhus, produced by the same *materies morbi*, but of greater virulence or larger amount, and influenced in its manifestation by constitutional and predisposing differences. These different varieties were attended with all grades of intensity, from the mildest grade of typhoid fever, in which the patient was scarcely sick enough to take his bed, to the gravest form of the same variety, in which the small and rapid pulse, the tympanitic abdomen, the frequent and involuntary alvine discharges attested the severity of the disease. The well marked cases of typhus were few in number, compared with the first variety, but the same difference in intensity was manifested, and so of the last most fearful form, spotted fever. Like the cases of typhus, this last variety afforded us but few cases compared with the first, but it showed the same difference in grade. In some cases the patient complaining of but little more than loss of appetite, with pain in back portion of head and neck, with general soreness and uneasiness of body. In others again the disease would be ushered in with the usual chill, the surface soon becoming covered with petechial spots, and the organic nervous centres becoming so severely shocked by the specific cause that death would come to the little sufferer's relief before it had passed many hours or days in this alarming condition. * * * *

The first two cases of spotted fever which made their appearance in our neighborhood were very violent, and both died in some forty-eight hours after the attack. They were brothers, aged respectively twelve and fourteen years. I did not see the cases, but learned from the physician who attended them that their cases manifested all the usual symptoms attributed to bad cases of spotted fever, as it has been witnessed in the different sections where it has prevailed of late. Such as severe pain in the occipital and nuchal regions, extreme soreness and sensitiveness of flesh and skin, dark colored eruption, delirium and great restlessness, with a hurried but feeble pulse, etc.

A few days after the death of these patients, I was called to see a little girl, aged about twelve years, who was taken with a chill, followed by moderate arterial re-action, pulse 120 and of moderate volume, great restlessness, throwing herself from side to side, severe pain in head, neck and back. In three hours from the time the chill ceased a general efforescence was thrown out over the whole body, of a measle-like character, which continued some twenty-four hours, becoming of a darker hue as it faded. Paralysis of right arm came on during first twelve hours, and continued some two days and recovered. After some two days of extreme suffering, patient gradually got better, and after an illness, very much like typhoid fever, of some two weeks' duration, she recovered.

Another case which came under my notice of a little girl, about same age as the first, after a period of two or three days of violence, in which the marked pain in head, neck and back, with raving delirium, extreme soreness of body, ecchymosed eruption, etc., were quite prominent, passed into a condition so much like our usual cases of typhus or typhoid fever, that it would have been taken for such and nothing more by one knowing nothing of its early history. Bleeding at nose and partial deafness were very common with our typhoid patients during the winter. These symptoms, particularly the deafness, was very marked and troublesome in this case. The child is having a very tedious convalescence. A low grade of fever, dry tongue, with sordes upon teeth and lips, continued soreness of flesh, great nervousness, almost complete deafness, slight acceleration of pulse at night, are the prominent symptoms which continued some two weeks after the violence of first few days passed over. She is now about the close of her fourth week, and just beginning to sit up.

A well marked case of this form of disease in the case of a man about thirty years of age, has been undei treatment by Dr. Clark, of our town, and after several days of alarming symptoms, in which the characteristic occipital and nuchal pain, the petechial eruption, raving delirium, soreness of flesh, etc., were very well defined, he passed into the same train of symptoms common to our typhoid patients—even the characteristic diarrhœa and tenderness on pressure, so common in typhoid fever, existed in his case.

In summing up these cases from the beginning to the close of our epidemic, I think the following points are apparent:

First, that we have had in the same epidemic well marked cases of typhoid, typhur and spotted fever: Secondly, that they were all produced by the *same* specific cause, and are but *varieties* of the same disease: and, Thirdly, that it is reasonable to treat them *all* on the same general plan of medication. * * * *

As to the specific cause producing this group of disease, the profession is agreed, I believe, that it is a blood poison. That the cases are *toxicological* certainly cannot be denied. Every symptom tells the story that a poison is circulating in the blood and disturbing the two fundamental properties of living structures—vital affinity and susceptibility. And also that the three classes, typhoid, typhus and spotted fever, being produced by the same morbific agent, are but varieties of the same topic disease, the dissimilarities owing to the *amount* or *virulence* of the poisonous agent, seemed to my mind true.

As to the plan of remedial treatment, it seems reasonable, as well as warranted by the most careful observation, that in all these varieties the two general indications are:

1st.—To destroy the blood poison and arrest the septic tendency of the fluids; and,

2d.—Assist in the elimination by keeping the emunctories fully open, especially the skin and kidneys.

The condition of the bowels in typhoid, the great innervation in typhus, and the *cerebro spinal lesion* in spotted fever, as important as they are, we consider but lesions developed in the course of the disease, and must be met as symytoms indicate. And in regard to the different agents used for this purpose, there will be a difference among practitioners.

But in regard to the first *two* indications, destroying the blood poison and securing its elimination, there ought, it occurs to me, be more harmony than there is. These are *uniform* conditions, alike in all cases. What will destroy the poison in *one* case, *as a rule*, will in all. And as a rule, what will eliminate in *one* case will in *all*. The *lesions* set up in the system in the course of the disease are ever varying with the constitutional differences, and must be met accordingly.

From late experiments made with the use of the sulphites of soda and lime in arresting the blood ferment in the eruptive fevers, they have been offered to us as promising success in filling the first indication in the disease under consideration. And so far as I have used the sulphite of soda in spotted fever, I must speak favorably of its use. I did not give it a trial in typhoid or typhus, but from its use in what of spotted fever I have had to treat, I think it promises much in this direction. How far can others speak of its use? or have they any thing by this time better? In my treatment of spotted fever, I relied on sulphite of soda and chlorate of potash for the first indication; on tinct. belladonna and counter irritation to arrest the engorgement of brain and spinal cord, with such use of tinct. cantharides, chlor. tinct. ferri, spts. nit. ether and ol terebinth, as seemed indicated. I was influenced in the use of tinct. belladonna from the result of Brown Sequard's experiments, and also seeing it recommended by Prof. N. S. Davis, of Chicago. And from its effects in my hands, I feel like giving it more extensive use if I should again be called to treat that or similar disease of those structures.—*Cincinnati Lancet.*

